# Model design for nonparametric phylodynamic inference and applications to pathogen surveillance

**DOI:** 10.1093/ve/vead028

**Published:** 2023-05-05

**Authors:** Xavier Didelot, Vinicius Franceschi, Simon D. W Frost, Ann Dennis, Erik M Volz

**Affiliations:** School of Life Sciences and Department of Statistics, University of Warwick, United Kingdom; Department of Infectious Disease Epidemiology, School of Public Health, Imperial College London, United Kingdom; Microsoft Research, USA; Department of Medicine, University of North Carolina, USA; Department of Infectious Disease Epidemiology, School of Public Health, Imperial College London, United Kingdom

**Keywords:** phylodynamics, effective population size, genomic epidemiology, HIV-1, SARS-CoV-2

## Abstract

Inference of effective population size from genomic data can provide unique information about demographic history and, when applied to pathogen genetic data, can also provide insights into epidemiological dynamics. The combination of nonparametric models for population dynamics with molecular clock models which relate genetic data to time has enabled phylodynamic inference based on large sets of time-stamped genetic sequence data. The methodology for nonparametric inference of effective population size is well-developed in the Bayesian setting, but here we develop a frequentist approach based on nonparametric latent process models of population size dynamics. We appeal to statistical principles based on out-of-sample prediction accuracy in order to optimize parameters that control shape and smoothness of the population size over time. Our methodology is implemented in a new R package entitled *mlesky*. We demonstrate the flexibility and speed of this approach in a series of simulation experiments and apply the methodology to a dataset of HIV-1 in the USA. We also estimate the impact of non-pharmaceutical interventions for COVID-19 in England using thousands of SARS-CoV-2 sequences. By incorporating a measure of the strength of these interventions over time within the phylodynamic model, we estimate the impact of the first national lockdown in the UK on the epidemic reproduction number.

## Introduction

Past fluctuation in the size of a population is reflected in the genealogy of a sample of individuals from that population. For example, under the coalescent model, two distinct lines of ancestry coalesce (i.e. find a common ancestor) at a rate that is inversely proportional to the effective population size at any given time (Kingman 1982; Griffiths and Tavare 1994; Donnelly and Tavare 1995). More coalescent events are therefore likely when the population size is small compared to when the population size is large. This causal effect of population size on genealogies can be reversed in an inferential framework to recover past population size dynamics from a given pathogen genealogy. This approach to inference of past demographic changes was first proposed 20 years ago (Pybus, Rambaut and Harvey 2000; Pybus et al. 2001; Strimmer and Pybus 2001) and has been fruitfully applied to many disease systems (Pybus and Rambaut 2009; Ho and Shapiro 2011; Baele et al. 2016).

Population size analysis is often performed within the Bayesian BEAST framework (Suchard et al. 2018; Bouckaert et al. 2019), which jointly infers a phylogeny and demographic history from genetic data. Here we focus on an alternative approach in which the dated phylogeny is inferred first, for example using treedater (Volz and Frost 2017), TreeTime (Sagulenko, Puller and Neher 2018), or BactDating (Didelot et al. 2018), and demography is investigated on the basis of the phylogeny. Although potentially less sensitive, this approach has the advantage of scalability to very large sequence datasets, which is why it has attracted increasing attention over the past few years (Didelot and Parkhill 2022). This post-processing approach also allows more focus on models and assumptions involved in the demographic inference itself as previously noted in studies following the same strategy (Lan et al. 2015; Karcher et al. 2017; Volz and Didelot 2018; Volz et al. 2020). However, some of the methodologies and results we describe here should be applicable in a joint inferential setting as well.

The reconstruction of past population size dynamics is usually based on a nonparametric model, since the choice of any parametric function for the past population size would cause restrictions and be hard to justify in many real-life applications (Drummond et al. 2005; Ho and Shapiro 2011). However, even if a nonparametric approach offers a lot more flexibility than a parametric one, it does not fully circumvent the question of how to design the demographic model to use as the basis of inference. For example, the *skygrid* model considers that the logarithm of the effective population size is piecewise constant, with values following a Gaussian Markov random field, in which each value is normally distributed around neighboring values and standard deviation determined by a smoothing hyperparameter (Gill et al. 2013). This model can be justified as an approximation to the *skyride* model in which the logarithm of the population size is allowed to change at each coalescent time following a Brownian motion (Minin, Bloomquist and Suchard 2008). Alternatively, the *skygrowth* model is a similar Gaussian Markov random field on the growth rate of the population size (Volz and Didelot 2018). Both models can be conveniently extended to explore the association between population size dynamics and covariate data (Gill et al. 2016; Volz and Didelot 2018).

The *skygrid*, *skygrowth*, or other similar models can be assumed when performing the inference of the demographic function, and the effect of this model choice has not been formally investigated. Furthermore, these nonparametric models require several model design choices which are often given little consideration in practice. This includes the number of pieces in the piecewise constant demographic function, the location of boundaries between pieces, and the prior expectation for the difference from one piece to another. All of these model design choices may have a significant effect on the inference results. Several previous studies have investigated some of these questions, and our study therefore represents an additional contribution to the growing body of research on this topic. Strimmer and Pybus (2001) used the Akaike information criterion (AIC) to choose the number and position of pieces in the demographic function. Parag and Donnelly (2020) compared this AIC with the Bayesian information criterion (BIC) and a frequentist generalization of both. On the other hand, Opgen-Rhein, Fahrmeir and Strimmer (2005) proposed a reversible jump Markov chain Monte Carlo to estimate the dimension and smoothing of the demographic function. Minin, Bloomquist and Suchard (2008) developed significance tests for the difference from one skyline piece to another, while Palacios and Minin (2013) attempted to reframe the smoothing selection problem within a Gaussian process framework. Gill et al. (2013) proposed the *skygrid* model described above, whereas the previous Bayesian skyline plot (Drummond et al. 2005) sampled across the locations of boundaries and used a different demographic function. The parameter controlling the smoothness of the population size function is usually assumed to have an arbitrary non-informative prior distribution in a Bayesian inferential setting (Minin, Bloomquist and Suchard 2008; Gill et al. 2013). As an exception to this, Faulkner et al. (2020) use weakly informative priors and present a method for automatically setting the hyperparameter for the global scale of the step increments. Most recently, Parag, Pybus and Wu (2022) developed metrics for choosing both the resolution and the smoothness based on how much information they contribute to effective population size estimates, and Bouckaert (2022) combined conjugate gamma priors on the effective population size with Markov chain Monte Carlo integration to implicitly perform the regularization.

Here we propose several statistical procedures to optimize these variables and implement them in a new R package entitled *mlesky*. In particular, we propose a frequentist statistical approach based on out-of-sample prediction accuracy in order to select the smoothness parameter. We tested the effect of these procedures on simulated datasets, where the correct demographic function is known and can be used to assess the relative accuracy of inference under various conditions. We applied our methodology to real data on HIV-1 in the USA and SARS-CoV-2 in England.

## Materials and methods

### Demographic Models

Let the demographic function $N_{\mathrm{e}}(t)$ denote the effective population size of a pathogen at time *t*. Let us consider that $N_{\mathrm{e}}(t)$ is piecewise constant with *R* pieces of equal length *h* over the timescale of interest. Let *γ*_*i*_ denote the logarithm of the effective population size in the *i*-th piece. In the *skygrid* model (Gill et al. 2013), the values of *γ*_*i*_ follow a Gaussian Markov random field, with the conditional distribution of $\gamma_{i+1}$ given *γ*_*i*_ equal to:


(1) $$ \gamma_{i+1} \sim \mathcal{N}(\gamma_i,h/\tau), $$

where *τ* is a precision parameter also known as the ‘smoothing’ parameter.

By contrast, the *skygrowth* model (Volz and Didelot 2018) is defined using the effective population size growth rates *ρ*_*i*_, which are assumed constant in each interval and are equal to:


(2) $$ \rho_i=\frac{\mathrm{exp}(\gamma_{i+1})-\mathrm{exp}(\gamma_i)}{h \mathrm{exp}(\gamma_i)}. $$

These growth rate values form a Gaussian Markov random field, with:


(3) $$ \rho_{i+1} \sim \mathcal{N}(\rho_i,h/\tau). $$

We also define a third model which we call *skykappa* based on the values *κ*_*i*_ of the second-order differences of the logarithm of the effective population size:


(4) $$ \kappa_i=(\gamma_{i+1}-\gamma_i)-(\gamma_i-\gamma_{i-1})=\gamma_{i+1}-2\gamma_i+\gamma_{i-1}. $$

Once again we consider a Gaussian Markov random field in which:


(5) $$ \kappa_{i+1} \sim \mathcal{N}(\kappa_i,h/\tau). $$

The *skykappa* model is a second-order random walk or second-order Gaussian Markov random field model. Faulkner et al. (2020) used the second-order random walk models extensively and called them GMRF-2 in the case of the standard Gaussian Markov random field as a random walk of order 2. Palacios and Minin (2013) used an integrated Brownian motion model, which is a continuous version of the second-order random walk, for testing prior sensitivity.

Dependency on known covariate time series can be easily incorporated into these models as previously described (Gill et al. 2016; Volz and Didelot 2018). Let there be a *m* × *p* matrix $X_{1:m,1:p}$ of *p* covariate measurements for each of *m* time points. Ideally, these time points would correspond to the *R* + 1 boundaries between pieces of the demographic function, but otherwise linear interpolation can be used to make it so. We model the effect of this covariate data as a modification of the expected change in the demographic variables defined above ($\gamma_i, \rho_i$, or *κ*_*i*_). For example, in the *skykappa* model (Equation 5), the kernel of the Markov random field becomes:


(6) $$ \kappa_{i+1} \sim \mathcal{N}( \kappa_i + (X_{i+1,1:p} - X_{i,1:p}) \beta , h/\tau), $$

where $\beta_{1:p}$ is a vector of coefficients for a linear model of the covariate data on the expected value of the stepwise differences $\kappa_{i+1}-\kappa_i$. Note in particular that if a term in the *β* vector is equal to zero, then this covariate measurement has no effect on the demographic function, so that testing the significance of covariate requires testing whether the corresponding value in the *β* vector is nonzero.

### Coalescent framework

Each of the models above defines a demographic function $N_{\mathrm{e}}(t)$ from which the probability of the genealogy $\mathcal{G}$ can be calculated as briefly described below. Let *n* denote the number of leaves in $\mathcal{G}$, let $s_{1:n}$ denote the dates of the leaves, and let $c_{1:(n-1)}$ denote the dates of the internal nodes. Let *A*(*t*) denote the number of extant lineages at time *t* in $\mathcal{G}$, which is easily computed as the number of leaves dated after *t* minus the number of internal nodes dated after *t*:


(7) $$ A(t)=\sum_{i=1}^n \mathbb{1}[s_i \gt t] - \sum_{i=1}^{n-1} \mathbb{1}[c_i \gt t]. $$

This quantity is important because in the coalescent model, each pair of lineages finds a common ancestor at rate $1/N_{\mathrm{e}}(t)$. Since there are $A(t)(A(t)-1)/2$ unordered pairs of lineages at time *t*, the total coalescent rate at time *t* is equal to:


(8) $$ \lambda(t)=\begin{cases} \frac{A(t)(A(t)-1)}{2N_{\mathrm{e}}(t)},& \text{if } A(t)\geq 2,\\ 0,& \text{otherwise.} \end{cases} $$

The full probability of the coalescent process is therefore computed as (Griffiths and Tavare 1994; Donnelly and Tavare 1995):


(9) $$ p(\mathcal{G}|N_{\mathrm{e}}(t))=\exp \left(-\int_{-\infty}^{\infty}\mathbb{1}[A(t)\geq 2] \frac{A(t)(A(t)-1)}{2N_{\mathrm{e}}(t)} \mathrm{d}t\right) \prod_{i=1}^{n-1} \frac{1}{N_{\mathrm{e}}(c_{i})}. $$

This computation is straightforward for the models considered here where the demographic function $N_{\mathrm{e}}(t)$ is piecewise constant. Finally, we can define the likelihood of the joint demographic/phylogenetic process as:


(10) $$ L=p(\mathcal{G},N_{\mathrm{e}}(t))=p(\mathcal{G}|N_{\mathrm{e}}(t))p(N_{\mathrm{e}}(t)). $$

This likelihood is the product of the probability of the coalescent process given in Equation 9 times the probability of the demographic function, which is determined by Equation 1, 3, or 5, depending on the model used.

### Selection of the precision parameter

The demographic models described above (*skygrid*, *skygrowth*, and *skykappa*) all rely on a precision parameter *τ*. The value of *τ* controls the amount of variance between consecutive values of the parameters used by each model. The selection of this parameter is therefore shaped by competing aims of optimizing the fit to observed data and maximizing explanatory power and avoidance of overfitting. In frequentist statistics, a standard approach to selecting smoothing parameters is to minimize the out-of-sample prediction error. For the problem of phylodynamic inference, Bayesian methods have predominated, and there have been few applications of cross-validation for model selection, although the use of such strategies in a hierarchical Bayesian setting has been considered (Duchêne et al. 2016). Here, we propose a novel strategy based on *k*-fold cross-validation where genealogical data are partitioned into *k* sets, *k* − 1 of which are used for fitting, and the last one is used for prediction. This procedure is equivalent to maximizing the following objective function:


(11) $$\begin{aligned} f(\tau) &= \prod_{j=1}^{k} p( \mathcal{G} \setminus X_j | \hat{N}_{\mathrm{e}}(X_j,\tau) ), \end{aligned}$$

where $\hat{N}_{\mathrm{e}}(X_j,\tau)$ is the maximum likelihood estimate of the demographic function $N_{\mathrm{e}}(t)$ on the partial data $X_j\subset \mathcal{G}$ and assume the precision parameter is *τ*. In this case $X_{j=1:k}$ represents a subset of the sample times and internal node times of the genealogy $\mathcal{G}$.

This is a standard formulation of the cross-validation method, but the implementation depends on how genealogical data are partitioned. We use the strategy of discretizing the coalescent probability (Equation 9) into intervals bordered by the time of nodes (leaves *s*_*i*_ plus internal nodes *c*_*i*_ of the tree) and the *R* − 1 times when the piecewise-constant $N_{\mathrm{e}}(t)$ function changes value. Given *R* − 1 change points, *n* leaves, and *n* − 1 internal nodes of $\mathcal{G}$, there are $R+2n-3$ intervals $(\iota_1, \cdots, \iota_{R+2n-3})$. Each cross-validation training set is formed by taking a staggered sequence of these intervals and collecting the genealogical data contained in each, so that $X_j = \{\iota_{a=1:R+2n-3} | (a+j-1)\ \mathrm{mod}\ k \neq 0 \}$. The cross-validation test sets are made of the remaining intervals, so that $\mathcal{G}\setminus X_j =\{\iota_{a=1:R+2n-3} | (a+j-1)\ \mathrm{mod}\ k = 0 \}$. For example, if *n* = 5, *R* = 4, and *k* = 3 we have $R+2n-3=11$ intervals denoted $(\iota_1, \cdots, \iota_{11})$. The training sets are $X_1=\{\iota_1,\iota_2,\iota_4,\iota_5,\iota_7,\iota_8,\iota_{10},\iota_{11}\}$, $X_2=\{\iota_1,\iota_3,\iota_4,\iota_6,\iota_7,\iota_9,\iota_{10}\}$, and $X_3=\{\iota_2,\iota_3,\iota_5,\iota_6,\iota_8,\iota_9,\iota_{11}\}$. The corresponding test sets are $\mathcal{G}\setminus X_1=\{\iota_3,\iota_6,\iota_9\}$, $\mathcal{G}\setminus X_2=\{\iota_2,\iota_5,\iota_8,\iota_{11}\},$ and $\mathcal{G}\setminus X_3=\{\iota_1,\iota_4,\iota_7,\iota_{10}\}$.

### Selection of the grid resolution

Before any of the nonparametric models described above can be fitted, the number *R* of pieces in the piecewise demographic function needs to be specified. Setting *R* too low may lead to an oversimplified output that does not capture all the information on past population changes suggested by the genealogy, whereas setting *R* too high can lead to overfitting.

We therefore propose to use well-established statistical methods to select the optimal value of *R*. First the model is fitted for multiple proposed values of *R*, and then for each output we compute the AIC, which is equal to:


(12) $$ \mathrm{AIC}_R=2R-2\mathrm{log}(L_R), $$

where *L*_*R*_ is the maximum value of the likelihood when using *R* pieces. The value of *R* giving the smallest value of $\mathrm{AIC}_R$ is selected. We also implemented the BIC, which is equal to:


(13) $$ \mathrm{BIC}_R=R \mathrm{log}(n-1)-2\mathrm{log}(L_R). $$

The AIC and BIC have been used for similar problems before, for example to generate the generalized skyline plot (Strimmer and Pybus 2001) and to select the number of knots in smoothing approaches such as B-splines (Malloy, Spiegelman and Eisen 2009).

### Simulation of testing data

In order to test the accuracy of our methodology, we implemented a simulator of coalescent genealogies given sampling dates and a past demographic function $N_{\mathrm{e}}(t)$, following a similar approach as previously used elsewhere (Adams, Murray and MacKay 2009; Palacios and Minin 2013; Karcher et al. 2017) and briefly outlined below. When the demographic function is constant, the simulation of coalescent genealogies is equivalent to simulating from a piecewise homogeneous Poisson process, in which the waiting times from one event to the next are exponentially distributed. To extend this to the situation where the demographic function is non-constant requires to simulate from an equivalent non-homogeneous Poisson process. The approach we used to achieve this is to consider a homogeneous Poisson process with a population size $N_{\mathrm{m}}$, which is lower than any value of $N_{\mathrm{e}}(t)$, i.e. $\forall t, N_{\mathrm{e}}(t) \geq N_{\mathrm{m}}$. We simulate this process using exponential waiting times, but filter an event happening at time *t* according to the ratio $N_{\mathrm{m}}/N_{\mathrm{e}}(t)$. Specifically, we draw $u \sim \mathrm{Unif}(0,1)$ and if $u\ltN_{\mathrm{m}}/N_{\mathrm{e}}(t)$ the event is accepted and otherwise rejected. The resulting filtered Poisson process simulates from the non-homogeneous Poisson process as required (Ross 2014). The disadvantage of this approach over other methods of simulations is that there may be many rejections if $N_{\mathrm{e}}(t)$ takes small values so that $N_{\mathrm{m}}$ needs to be small too. However, the efficiency of simulation is not important for our purpose here, and this method has the advantage to avoid the computation of integrals on the $N_{\mathrm{e}}(t)$ function which other methods would require.

To measure $N_{\mathrm{e}}(t)$ estimation accuracy through time across different demographic models and sample sizes, 500 dated phylogenies were simulated with a total of $n \in \{100,200\}$ leaves sampled uniformly at regular intervals between 2000 and 2020, which are also represented as the times to the most recent sample -20 to 0. Additionally, constant and variable (sinus and bottleneck) demographic functions were applied. Since the $N_{\mathrm{e}}(t)$ for the constant and bottleneck functions do not have a characteristic timescale (one change in $N_{\mathrm{e}}(t)$ at maximum), we let the algorithms described above select *R* (see Methods section ‘Selection of the grid resolution’) and *τ* (see Methods section ‘Selection of the precision parameter’) for all trees. For the sinus function, which has a period of 2*π*, we have used a fixed *R* based on prior information (*R* = 30) and the cross-validation method to choose *τ* as for the other functions.

Coverage probabilities, defined as the proportion of samples for which the known population parameter is contained in the confidence interval according to the parametric bootstrap procedure (see Methods section ‘Implementation’), were calculated for each time point and summarized over the entire time axis. Since the confidence interval is of 95 per cent, we would expect around 475 of the 500 replicates to contain the true $N_{\mathrm{e}}(t)$ value for each time point and the overall coverage probability to be 0.95. Finally, mean absolute error (MAE) and root mean squared error (RMSE) were computed for the $N_{\mathrm{e}}(t)$ maximum likelihood estimate for each simulation and summarized over all simulations. Importantly, we discarded the simulated trees in which the optimal solution for grid resolution was $R \leq 2$ without replacement. This choice is anchored in subsequent considerations (see Methods section ‘Selection of the grid resolution’) about lack of power to retrieve all potential fluctuations on past population changes. Coverage probability, MAE, and RMSE plots were compared considering the three different demographic functions implemented by *mlesky* and sample sizes defined above.

Since the time of the pieces (knots) of the demographic function are variable across different simulated phylogenies, we defined a common time axis based on linear interpolation of time and $N_{\mathrm{e}}(t)$ estimates using the *approx* function from the *stats* package (R Core Team 2022) and getting the most recent first time of the pieces, as well as the older of the latest piece times across all simulations as respective boundaries for this common time axis. Then we define the new number of pieces of the unique time axis by dividing the total quantity of pieces across all 500 simulations by the amount of simulated trees and make their respective time points equally spaced. Using this approach, we could obtain comparable $N_{\mathrm{e}}(t)$ estimates across different simulations.

### Implementation

We implemented the simulation and inference methods described in this paper into a new R package entitled *mlesky* which is available at https://github.com/emvolz-phylodynamics/mlesky. The optimization of the demographic function makes use of the quasi-Newton Broyden–Fletcher–Goldfarb–Shanno (BFGS) method implemented in the optim command (Nash 2014). Confidence intervals (95 per cent) can be computed using (a) a standard bootstrap procedure if multiple samples from the bootstrap distribution of the ML phylogeny can be provided or (b) a parametric bootstrap procedure whereby coalescent trees are simulated conditional on the ML estimated of $N_e(t)$ and known sample times (see Methods section ‘Simulation of testing data’). If multiple CPU cores are available, these resources are exploited within the procedure of selection of the smoothing parameter where the computation can be split between the different cross values in the cross-validation. Multicore processing is also applied in the procedure of selection of the grid resolution where computation can be split between different values of the resolution parameter *R*. All the code and data needed to reproduce our results on simulated and real datasets is available at https://github.com/mrc-ide/mlesky-experiments.

## Results

### Application to simulated phylogenies with constant population size

**Figure 1.**
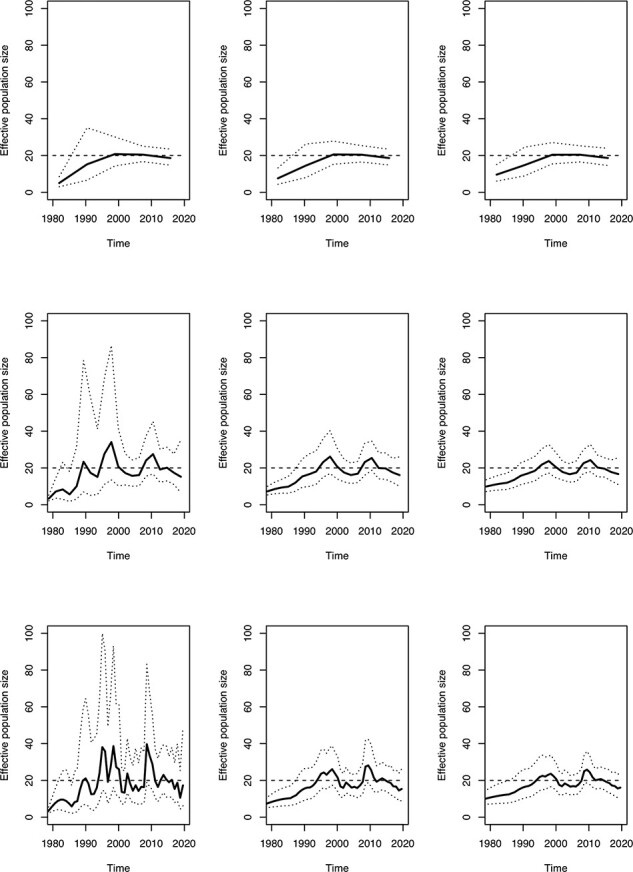
Result on a simulated phylogeny under a constant demographic function using the skygrid model, from top to bottom $R=5,20,50$ and from left to right $\tau=1,10,20$. The dashed line represents the correct function $N_{\mathrm{e}}(t)=20$.

A dated phylogeny was simulated with 200 leaves sampled at regular intervals between 2000 and 2020 and a constant past population size function $N_{\mathrm{e}}(t)=20$ (Fig. S1). To illustrate the importance of the resolution *R* and precision *τ* parameters, we inferred the demographic function under the *skygrid* model (cf Equation 1) for a grid of values with $R \in \{5,20,50\}$ and $\tau \in \{1,10,20\}$ (Fig. 1). The equivalent analyses under the *skygrowth* model (Equation 3) and the *skykappa* model (Equation 5) are shown in Figs. S2 and S3, respectively. The results look quite different depending on the parameters used, and in particular when *R* is large and *τ* is small, fluctuations in the population size are incorrectly inferred. When applying the AIC procedure to this dataset, the correct value of *R* = 1 was inferred for which the parameter *τ* becomes irrelevant. In these conditions the effective population size was estimated to be 19.65 with confidence interval ranging from 17.10 to 22.57, which includes the correct value of 20 used in the simulation.

After simulating 500 dated phylogenies and applying a constant past demographic function $N_{\mathrm{e}}(t)=20$, we attempted to reconstruct the demographic function based on the phylogeny under the three models *skygrid*, *skygrowth*, and *skykappa* described in Equations 1, 3, and 5, respectively. Regarding coverage probabilities (i.e. the probability that estimated confidence intervals given by *mlesky* cover the constant $N_{\mathrm{e}}(t)=20$ function in this case), all sample sizes and demographic models kept near entire coverage over time as expected (Table 1). Moreover, RMSE estimates were lower for *n* = 200 in comparison with *n* = 100, except for the *skygrid* model that presented more extreme error values (Fig. S4). The *skykappa* and *skygrowth* models performed very similarly for both sample sizes (Table 2 and Fig. S4).

**Table 1.** Coverage probabilities over time for the constant demographic function across 500 simulated phylogenies considering different sample sizes ($n \in \{100,200\}$)

	*n = 200*			*n = 100*		
Time	*skykappa*	*skygrid*	*skygrowth*	*skykappa*	*skygrid*	*skygrowth*
‒26.241	0.848	0.936	0.860	0.859	0.922	0.855
‒24.711	0.878	0.942	0.888	0.883	0.926	0.881
‒23.181	0.912	0.944	0.908	0.912	0.928	0.912
‒21.651	0.944	0.950	0.934	0.942	0.948	0.938
‒20.121	0.960	0.958	0.958	0.958	0.948	0.964
‒18.592	0.968	0.962	0.972	0.964	0.956	0.970
‒17.062	0.976	0.966	0.974	0.970	0.954	0.974
‒15.532	0.980	0.978	0.988	0.972	0.950	0.974
‒14.002	0.980	0.972	0.980	0.972	0.946	0.970
‒12.472	0.980	0.970	0.980	0.980	0.952	0.980
‒10.943	0.980	0.970	0.986	0.980	0.948	0.978
‒9.413	0.982	0.970	0.988	0.974	0.950	0.974
‒7.883	0.980	0.968	0.980	0.976	0.946	0.978

**Table 2.** RMSE mean, median, and IQR estimates across the 500 simulated phylogenies for the constant demographic function $N_{\mathrm{e}}(t)=20$ considering different sample sizes ($n \in \{100,200\}$)

	*n = 200*			*n = 100*		
RMSE	*skykappa*	*skygrid*	*skygrowth*	*skykappa*	*skygrid*	*skygrowth*
Mean	2.460	4.663	2.521	2.678	2.107	2.749
Median	2.067	1.323	2.066	2.453	1.684	2.481
IQR	1.632	1.414	1.599	2.054	1.821	2.057

### Application to simulated phylogenies with varying population size

Subsequently, we simulated 500 dated phylogenies with the same scheme of leaf number and dates as previously defined, but now using a demographic function $N_{\mathrm{e}}(t)$ that was sinusoidal with minimum 2 and maximum 22 and period 6.28 years. Figure S5 shows an example of both the demographic sinus function used and the resulting simulated phylogeny and Fig. S6 gives an example of inference using the three models.

**Figure 2.**
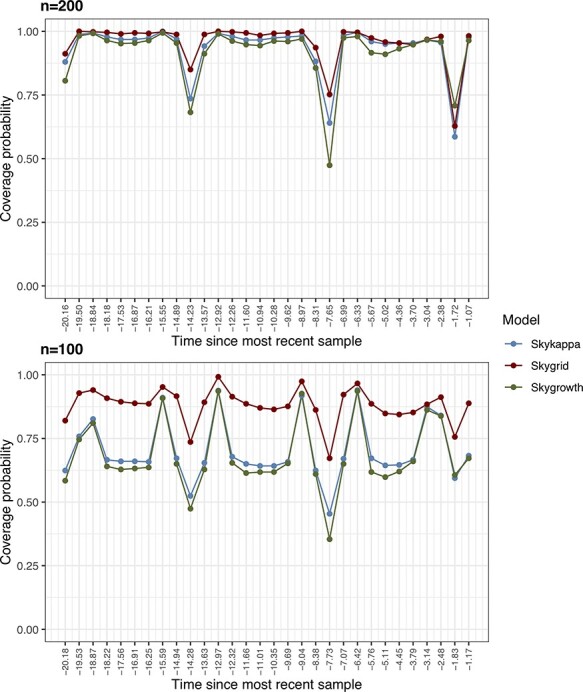
Coverage probabilities over time for the sinusoidal demographic function across 500 simulated phylogenies considering different sample sizes ($n \in \{100,200\}$) and colored by demographic model

Remarkably, the coverage probabilities for both sample sizes suffered from three main drops, each approximately occurring around the sinusoidal function period (6.28 years). The *skygrid* model performs slightly better than the other models for *n* = 200 and is significantly superior for *n* = 100 (Fig. 2). As expected, higher sample size was associated with lower RMSE values (Fig. S7). Despite RMSE estimates similar across models, the IQR for *skygrowth* and *skykappa* when *n* = 100 is >3, showing that error estimates are more spread out in these cases (Table 3). Figure S8 illustrates the effect of optimizing the value of *τ* using the new cross-validation procedure compared to several fixed values.

**Table 3.** RMSE mean, median, and IQR estimates across the 500 simulated phylogenies for the sinusoidal demographic function considering different sample sizes ($n \in \{100,200\}$)

	*n = 200*			*n = 100*		
RMSE	*skykappa*	*skygrid*	*skygrowth*	*skykappa*	*skygrid*	*skygrowth*
Mean	3.311	3.481	3.7	5.381	4.768	5.802
Mean	3.134	3.363	3.475	4.817	4.496	5.449
IQR	1.223	1.036	1.411	3.598	1.575	3.236

One situation in which all three models are expected to perform poorly is when there are sudden changes to the demographic function (Palacios and Minin 2013). To exemplify this, we simulated another set of 500 dated phylogeny with the same scheme of leaf number and dates as before, but using a bottleneck function for $N_{\mathrm{e}}(t)$ which was equal to 10 at all times except between 2005 (i.e. -15 years before most recent sample) and 2010 (i.e. -10 years before most recent sample) when it was equal to 1. An example is shown in Fig. S9.

**Figure 3.**
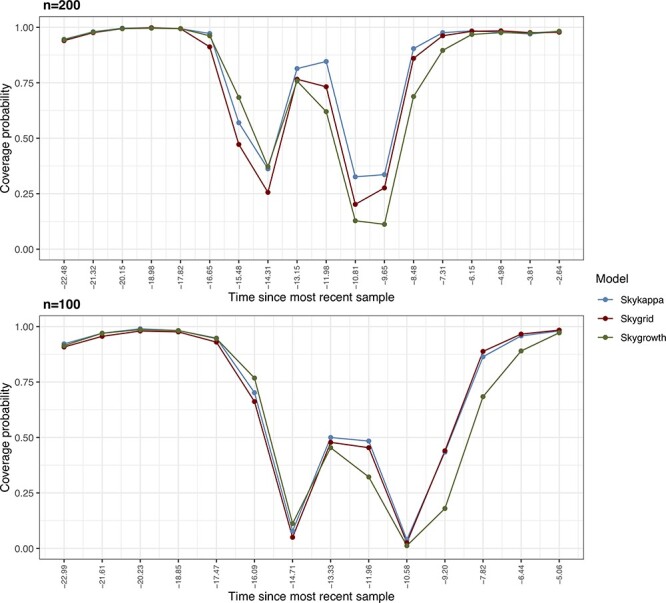
Coverage probabilities over time for the bottleneck demographic function across 500 simulated phylogenies considering different sample sizes ($n \in \{100,200\}$) and colored by demographic model

Under the bottleneck simulation scenarios, all models performed well for the time where the $N_{\mathrm{e}}(t)$ reproduced a constant function ($N_{\mathrm{e}}(t)=10$), but badly when the abrupt change (bottleneck event) to $N_{\mathrm{e}}(t)=1$ was reached. In the middle of the bottleneck interval there is also a noticeable improvement. Different models performed similarly in this case, even though there are some minor coverage probability peaks favoring the new *skykappa* model. Importantly, in the higher sample size scheme (*n* = 200), the $N_{\mathrm{e}}(t)$ estimates are covered in around 25 per cent of the simulations in the bottleneck event boundaries, whereas there are points with zero coverage for the lower sample size (*n* = 100), demonstrating that higher sample sizes can mitigate major estimation errors when the population process generating the data go against the priors of the employed demographic models (Fig. 3). RMSE estimates were slightly higher for the *skygrid* model with *n* = 100 but similar in the remaining scenarios (Fig. S10).

Collectively, these results suggest that when the precision parameter is optimized using the cross-validation method, the choice between these three models becomes less important. However, in practice, the choice of using one model rather than another is sometimes guided by the presence of covariate data and whether these are expected to correlate with the effective population size directly or some other function of it such as the population growth rates (Gill et al. 2016; Volz and Didelot 2018).

### Application to simulated phylogeny with covariate data

Finally, we used simulations to test our procedure for the analysis of association between demography and covariate data. An example is shown in Fig. S11 where the covariate data follows a simple function in order to create a boom and bust dynamic (Fig. S11A). The growth rate of the population is equal to this function times a multiplicative factor. From this growth rate we compute the effective population size function over time (Fig. S11B) and simulate a phylogenetic tree as previously, with 200 leaves sampled at regular intervals between 2000 and 2020 (Fig. S11C). We then analyzed this simulated phylogeny alongside the covariate data and found in this case an association with coefficient *β* = 0.44. We repeated this procedure 100 times with values of the multiplicative factor varying from zero (in which case the growth rate is constant equal to 0 and there is no association with the covariate) to nine times the factor used for Fig. S11. The results are summarized in Fig. S12. As expected, we found that as the multiplicative factor increases, the coefficient of association *β* between growth rate and the covariate increases, and that the association becomes zero when the multiplicative factor is zero.

### HIV-1 in the USA

To examine how effective population size is related to independent estimates of incidence and prevalence we investigated a dataset based on HIV-1 in North Carolina, USA. Several factors related to HIV epidemiology and the natural history of HIV infection may cause the relationship between HIV prevalence and *N*_*e*_ to be complex: The rate of diagnosis and treatment has increased over time. HIV infection leads to a treatable chronic infection resulting in low mortality. While prevalence has increased in the recent past due to reduced mortality, incidence has decreased, and a growing proportion of the infected population receives antiretroviral therapy and has suppressed viral loads. The frequency of transmission of HIV is typically concentrated in the early period (first year) of HIV infection because of higher viral loads, lower probability of being diagnosed and treated, and fluctuating risk behavior (Romero-Severson et al. 2015).

In Dennis et al. (2021) a dated phylogenetic tree was estimated using treedater (Volz and Frost 2017) based on 1,850 HIV-1 partial pol sequences sampled from North Carolina between 1997 and 2019. An estimate of new infections per year (denoted $\iota(t)$) and an estimate of the number of people living with HIV (denoted $\pi(t)$) in North Carolina were reported by the US Centers for Disease Control for the period 2010–2019 (Linley et al. 2019). We fit a skygrid model to these data, estimating the smoothing parameter by 5-fold cross-validation (which took approximately 30 s on a standard laptop computer) and estimating CIs with parametric bootstrap (which took approximately 90 s). Three covariates were considered:

1.
$\mathrm{log}(N_e(t))$ was modeled as proportional to $\mathrm{log}(\pi(t))$;
2.
$\mathrm{log}(N_e(t))$ was modeled as proportional to $\mathrm{log}(\iota(t))$;
3.
$\mathrm{log}(N_e(t))$ was modeled as proportion to $\nu(t) = \mathrm{log}(\pi(t)^2/\iota(t)) $.

This final formulation was derived as the asymptotic behavior of *N*_*e*_ in a population with variable incidence and prevalence. During periods where there is a stable relationship between incidence and prevalence (e.g. during exponential growth) there is a linear relationship between $N_e(t)$ and $\pi(t)$. Skygrid analysis showed that neither incidence nor prevalence had a significant association with *N*_*e*_. A highly significant association was seen for $\nu(t)$, with a coefficient $\beta_\nu = 2.05$ (95 per cent CI: 1.05–3.56).

### COVID-19 in England

In order to demonstrate the ability of the *mlesky* model to estimate the impact of public health interventions, we analyzed time-scaled phylogenies which were previously estimated for the B.1.1.7 (Alpha) SARS-CoV-2 lineage (Volz et al. 2021). In response to growing case numbers resulting from B.1.1.7, a national lockdown was implemented on 5 January 2021, resulting in a large decrease in human mobility outside of households. We combined phylogenetic data with information about human mobility collected from smartphone location tracking and publicly released by Google (Google LLC 2022) in the period of 1 November 2020 to 13 February 2021. We focus on the metric describing smartphone presence in transit stations, which is reported as a difference from historic baseline levels. We hypothesize that the decline in mobility and concomitant decline in incidence will be reflected by a drop in the growth rate of $N_{\mathrm{e}}$ and *mlesky* will estimate the strength of the association.

Effective population size may not decline immediately following lockdown since transmission can continue in some settings (households and hospitals) while transmission is heavily curtailed in the community. This can produce a lag between mobility metrics based on public transport attendance and the decline in transmissions. We investigated the time dependency of the association by first smoothing the mobility metrics (smooth.spline in R with 5-fold cross-validation) and then time-shifting the metric by between -15 and +36 days. For each shifted time series, we fit *mlesky* under a skygrid model with the shifted mobility metric as a single covariate. This was repeated for 500 time-scaled phylogenies, each reconstructed from 3,000 B.1.1.7 sequences. The running time for each lag value was less than 3 min on a standard laptop computer.

**Figure 4.**
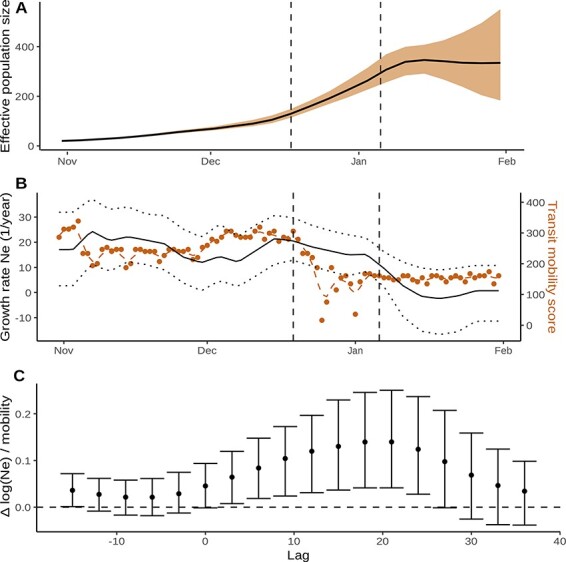
The epidemiological trajectory of SARS-CoV-2 lineage B.1.1.7 in England during spring 2020 inferred using *mlesky* and 3000 SARS-CoV-2 sequences. Dashed lines show dates (December 19, 2020 and January 6, 2021) when NPIs were implemented in England. (A) Effective population size $N_{\mathrm{e}}(t)$ through time. (B) Lines(black) show growth rate (1/year) in $N_{\mathrm{e}}(t)$ corresponding to panel A. Points (orange, right axis) show the human mobility score over time. (C) Estimated coefficient and 95 per cent confidence interval of the human mobility score on effective population size computed using *mlesky*. The mobility time series is shifted by a lag shown on the x axis.

Figure 4A shows the estimated effective population size through time, which peaked on 14 January 2021. The growth rate of effective size versus the mobility metric is shown in Fig. 4B. Note that human mobility declined precipitously in the period preceding lockdown with increasing awareness of B.1.1.7 and the end of the Christmas holiday. We find that human mobility has a large and significant impact on growth rate of $N_{\mathrm{e}}$; however, this effect is only apparent in the time-shifted data. The time lag showing the strongest association is +21 days (Fig. 4C).

## Discussion

Nonparametric phylodynamic inference of population size dynamics is usually carried out in a Bayesian framework (Drummond et al. 2005; Minin, Bloomquist and Suchard 2008; Gill et al. 2013). Here we presented methods for performing such inference in a frequentist setting with a particular view toward model selection and avoiding over-fitting. Optimal smoothing can be obtained in a natural way using standard cross-validation methods, and the optimal resolution of the discretized demographic function is achieved using the well-established AIC. This approach can be advantageous when prior distributions are difficult to design or results are sensitive to arbitrarily chosen priors. Methods based on likelihood maximization are also fast and scalable to datasets much larger than is conventionally studied with Bayesian methods, and the selection of smoothing parameters does not require arbitrarily chosen hyperparameters. Conventional AIC metrics also alleviate the difficulty of model selection. In most of our simulations, we find relatively little difference in our estimates when parameterizing the model in terms of $\log(N_{\mathrm{e}}(t))$ (Equation 1), the growth rate of $N_{\mathrm{e}}(t)$ (Equation 3), or the second-order variation of $\log(N_{\mathrm{e}}(t))$ (Equation 5), as long as the precision parameter *τ* for each model is optimized as we proposed.

Our methodology assumed that a dated phylogeny has been previously reconstructed from the genetic data. It is therefore well suited for the post-processing analysis of the outputs from *treedater* (Volz and Frost 2017) or *TreeTime* (Sagulenko, Puller and Neher 2018). A key assumption of our method, as with its Bayesian counterparts, is that all samples in the phylogeny come from a single population ruled by a unique demographic function. To ensure that this is indeed the case, complementary methods are emerging that can test for the presence or asymmetry or hidden population structure in dated phylogenies (Dearlove and Frost 2015; Volz et al. 2020). Conversely, if multiple phylogenies follow the same demographic dynamic, they can be analyzed jointly to provide a more precise reconstruction of the demographic function and epidemiological parameters (Xu et al. 2019), and our software implementation is able to perform such a joint analysis when appropriate. It should be noted that Bayesian phylogenetics is also increasingly concerned with the adequacy of the phylodynamic model used (Duchene et al. 2019) and has made considerable improvements in scalability over the past few years (Fisher et al. 2022).

Past variations in the effective population size of a pathogen population can reveal key insights into past epidemiological dynamics and help make predictions about the future. It is important to note that the effective population size is not generally equal to or even proportional to the number of infections over time (Volz et al. 2009; Dearlove and Wilson 2013). On the other hand, the growth rate of the effective population size can be used to estimate the basic reproduction number over time *R*(*t*) (Wallinga and Lipsitch 2007; Volz, Koelle and Bedford 2013; Volz and Didelot 2018) as we used in our application to COVID-19 in England. Having good estimates of this quantity is especially important for assessing the effect of infectious disease control measures (Fraser 2007), and phylodynamic approaches provide a useful complementary approach to more traditional methods of estimation based on case report data (Cori et al. 2013).

## Supplementary Material

vead028_Supp
