# Supplementary material for: Model design for nonparametric phylodynamic inference and applications to pathogen surveillance
Source: Virus Evol. 2023 May 5;9(1):vead028. doi: 10.1093/ve/vead028 (PMC10205094; doi:10.1093/ve/vead028)
Supplement: vead028_Supp [file vead028_supp.zip › suppl_data/supmat.pdf]

Supplementary Material

Model design for non-parametric phylodynamic inference and applications to pathogen surveillance

Xavier Didelot[1,*], Vinicius Franceschi[2], Simon D.W. Frost[3], Ann Dennis[4] and Erik M Volz[2]

[1] School of Life Sciences and Department of Statistics, University of Warwick, United Kingdom

[2] Department of Infectious Disease Epidemiology, School of Public Health, Imperial College London, United Kingdom

[3] Microsoft Research, USA

[4] Department of Medicine, University of North Carolina, USA

[*] Corresponding author. Tel: 0044 (0)2476 572827. Email: xavier.didelot@gmail.com

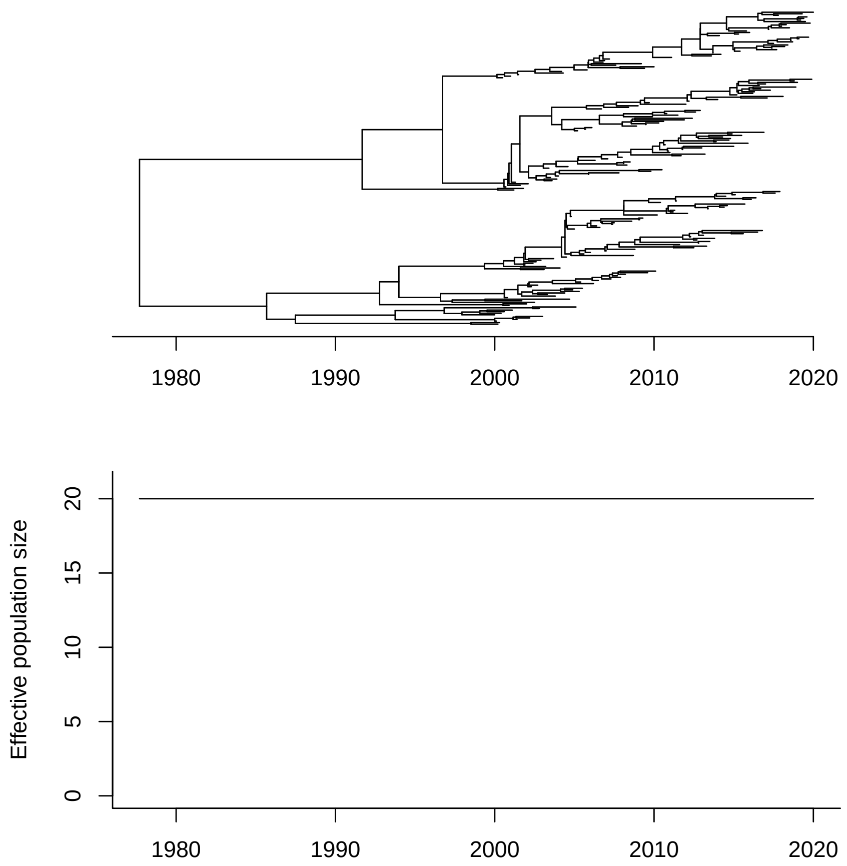

Figure S1: Simulated phylogeny using a constant demographic function.

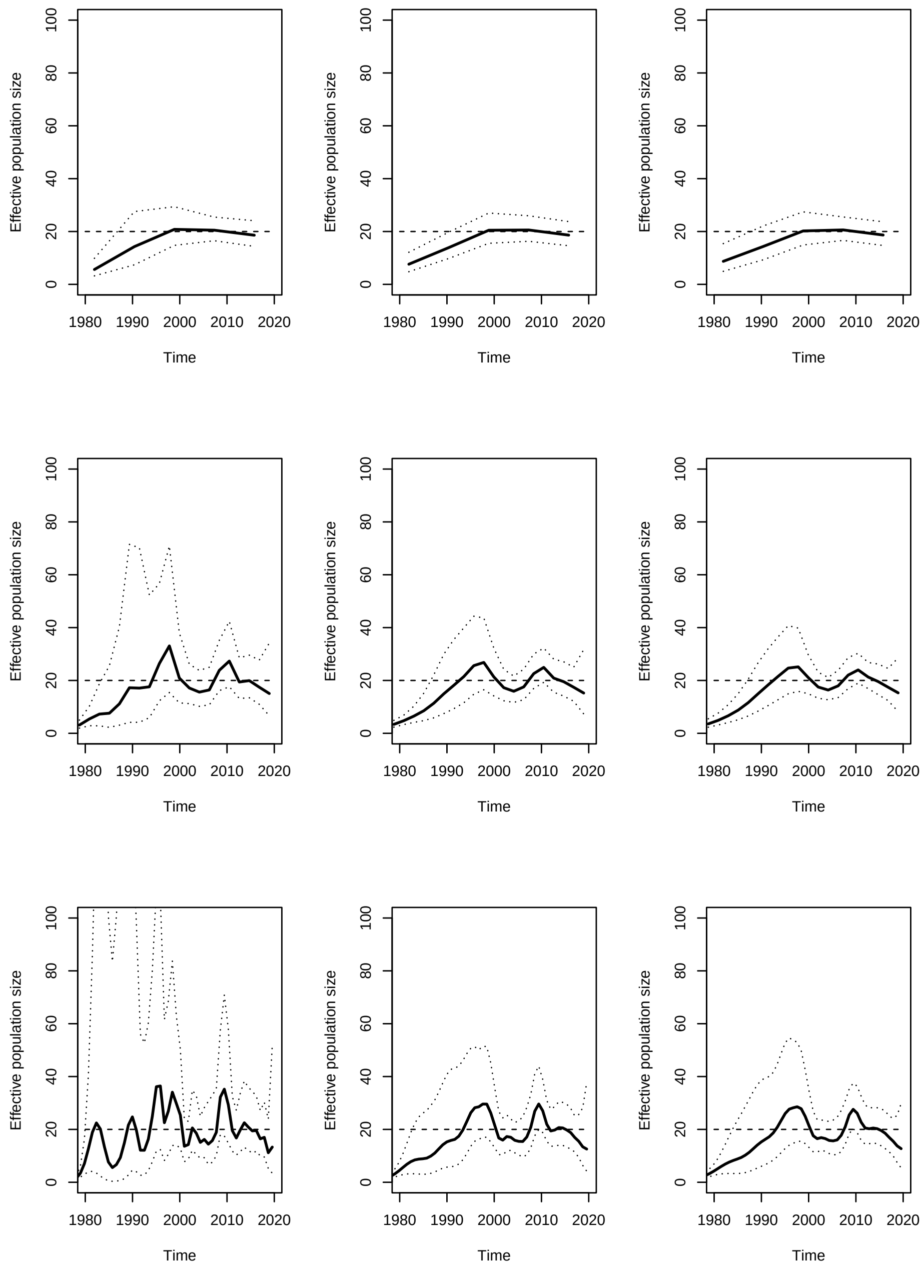

Figure S2: Result on a simulated phylogeny under a constant demographic function using the skygrowth model, from top to bottom $R = 5, 20, 50$ and from left to right $\tau = 1, 10, 20$. The dashed line represents the correct function $N_{\mathrm{e}}(t) = 20$.

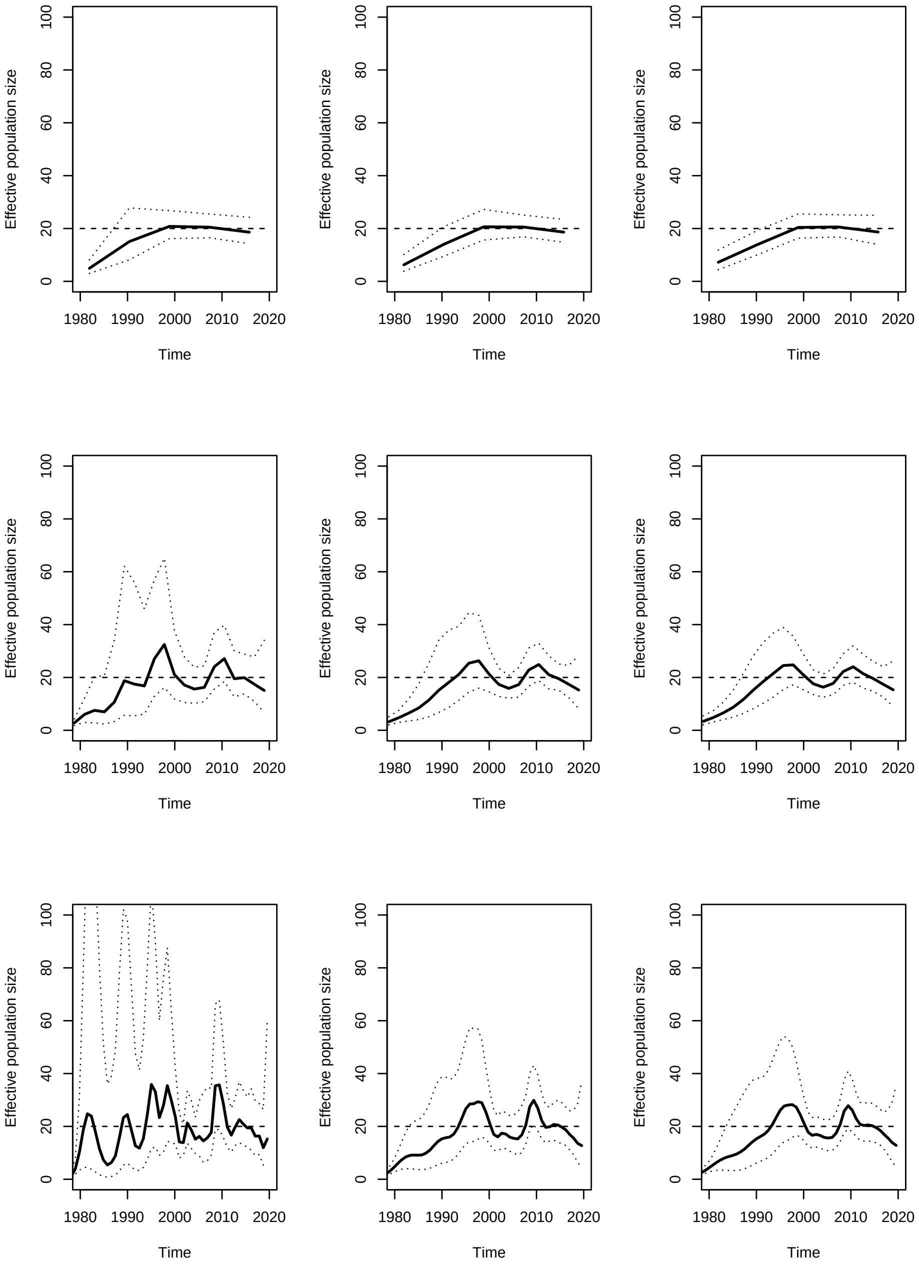

Figure S3: Result on a simulated phylogeny under a constant demographic function using the skykappa model, from top to bottom $R = 5, 20, 50$ and from left to right $\tau = 1, 10, 20$. The dashed line represents the correct function $N_{\mathrm{e}}(t) = 20$.

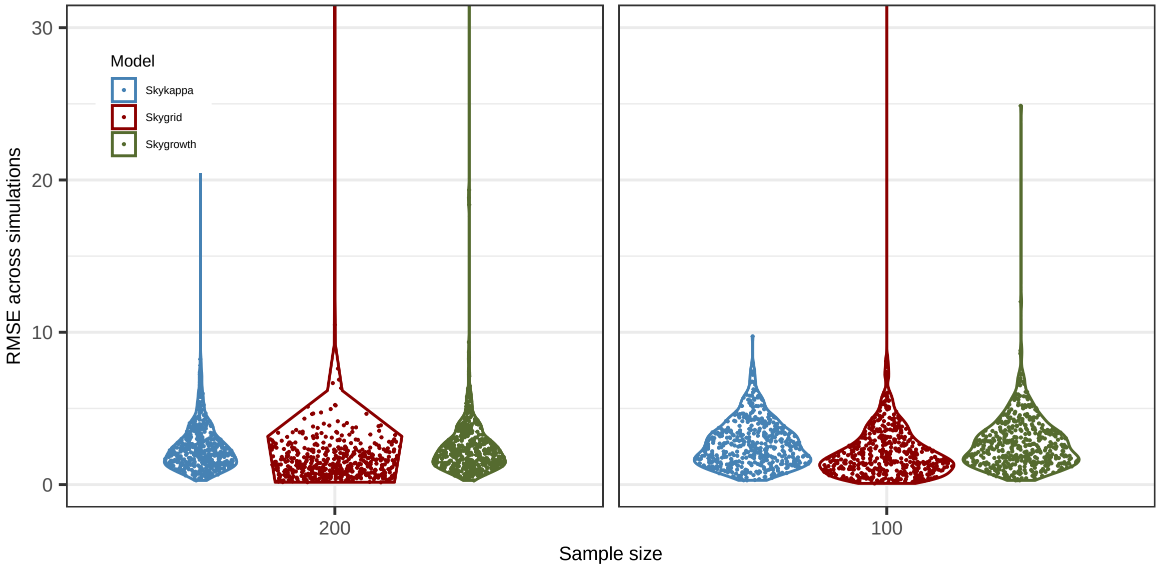

Figure S4: RMSE estimates for the constant demographic function $N_{\mathrm{e}}(t) = 20$ across 500 simulated phylogenies considering different sample sizes ($n \in \{100, 200\}$) and colored by demographic model

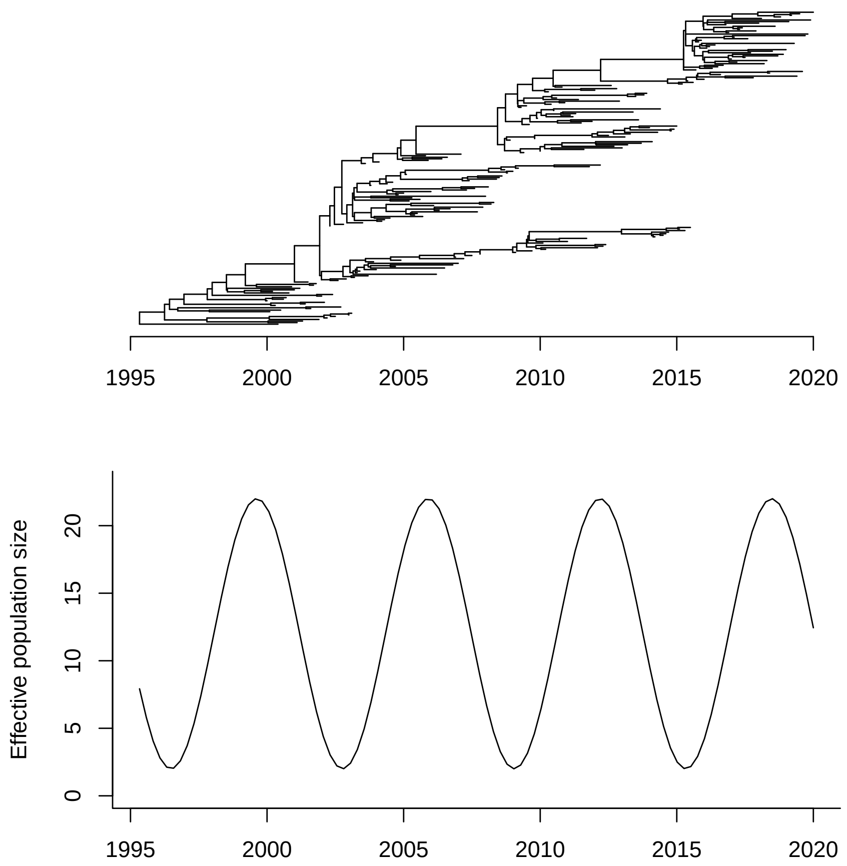

Figure S5: Simulated phylogeny using a sinusoidal demographic function.

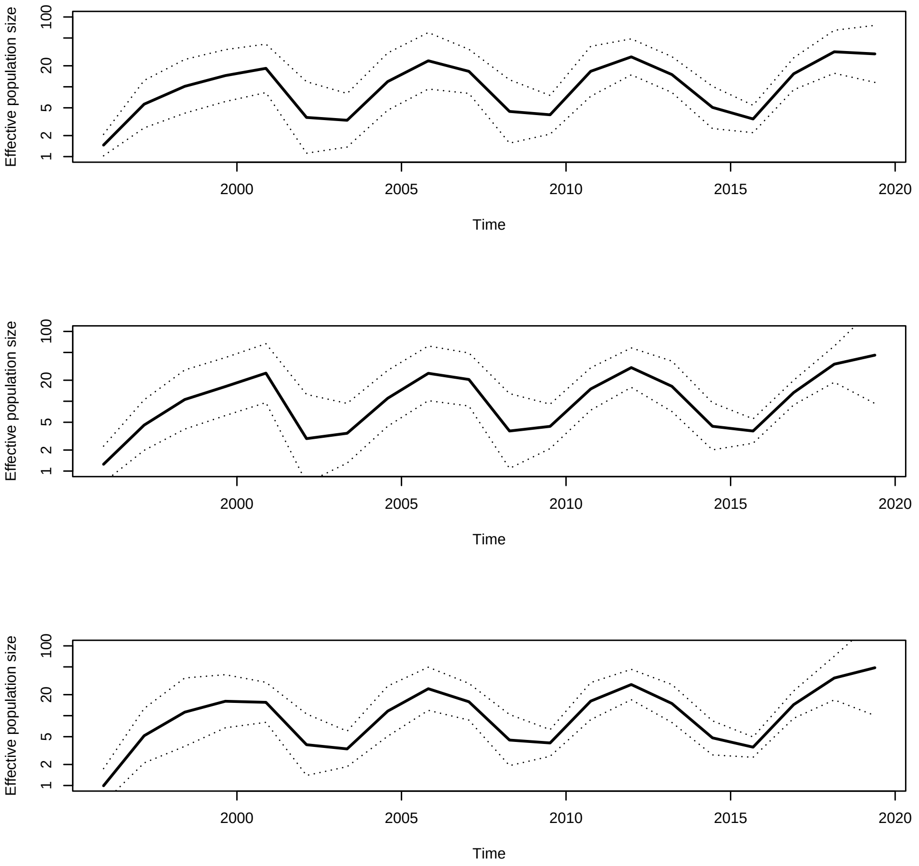

Figure S6: Result of applying the three different models (from top to bottom, *skygrid*, *skygrowth* and *skykappa*) to the phylogeny shown in Figure S5 which was simulated using a sinusoidal demographic function.

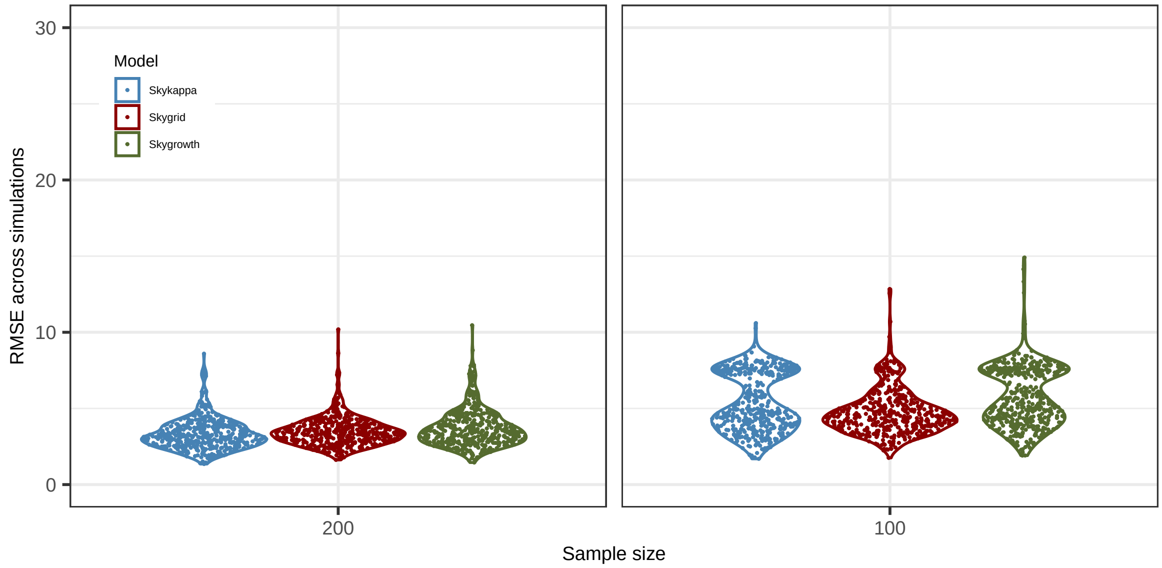

Figure S7: RMSE estimates for the sinusoidal demographic function across 500 attempted simulated phylogenies considering different sample sizes ($n \in \{100, 200\}$) and colored by demographic model

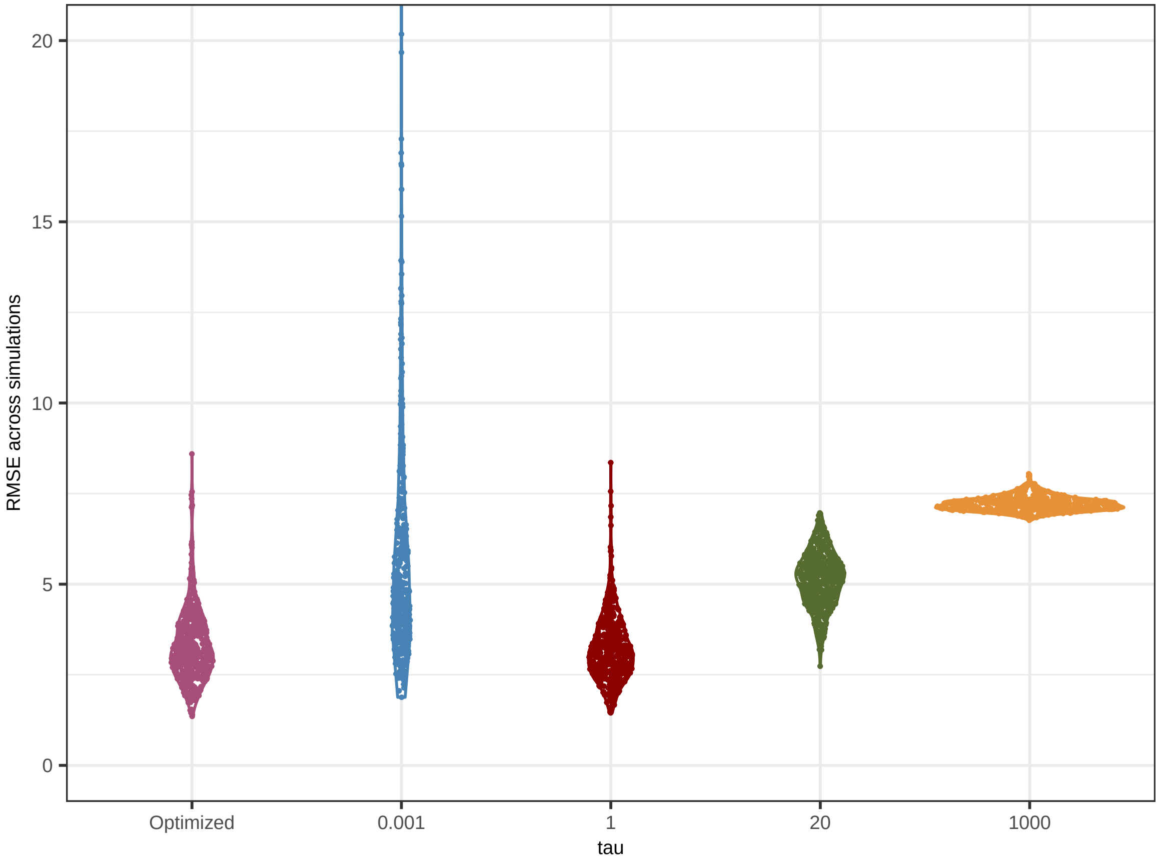

Figure S8: RMSE estimates for the sinusoidal demographic function across 500 attempted simulated phylogenies using $n = 200$ leaves and the newly proposed *skykappa* demographic model. τ was fixed for all simulations on values displayed on the horizontal axis, except when "Optimized" using the described cross-validation strategy.

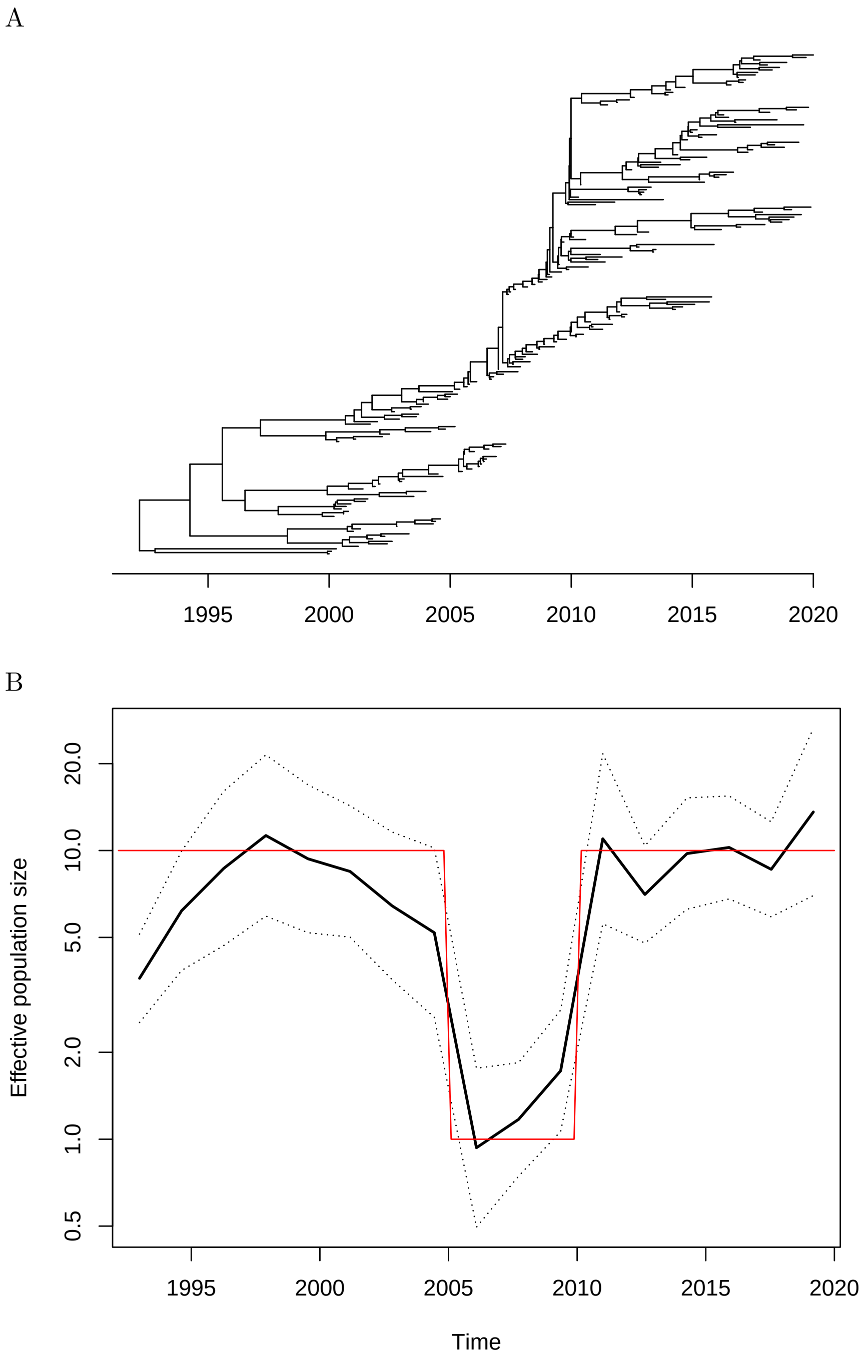

Figure S9: Demographic function (A) and phylogeny (B) for a simulated dataset under a bottleneck model.

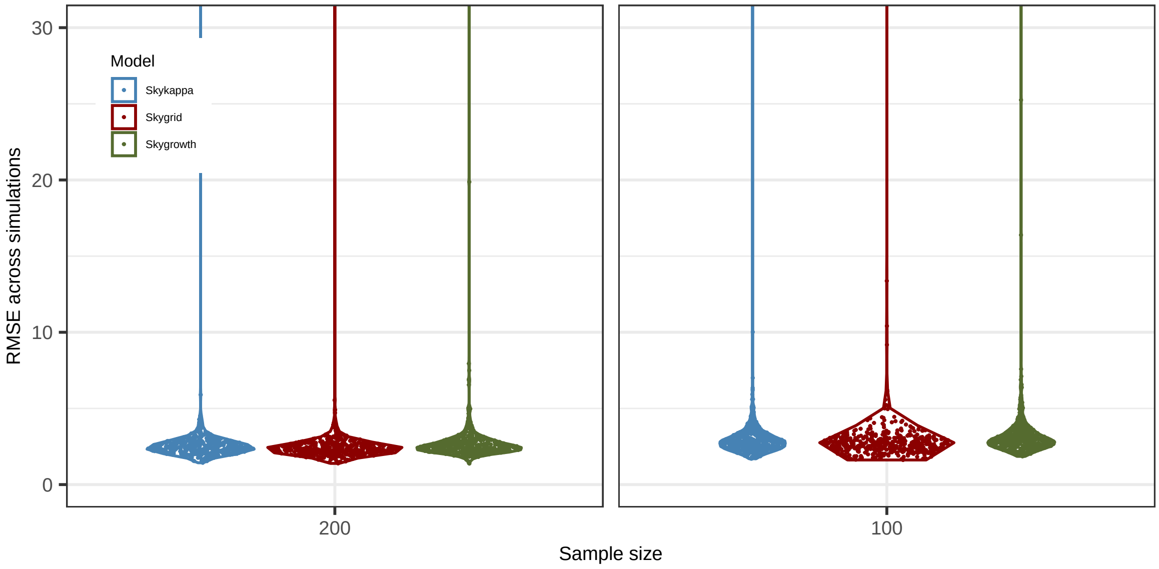

Figure S10: RMSE estimates for the bottleneck demographic function across 500 simulated phylogenies considering different sample sizes ($n \in \{100, 200\}$) and colored by demographic model

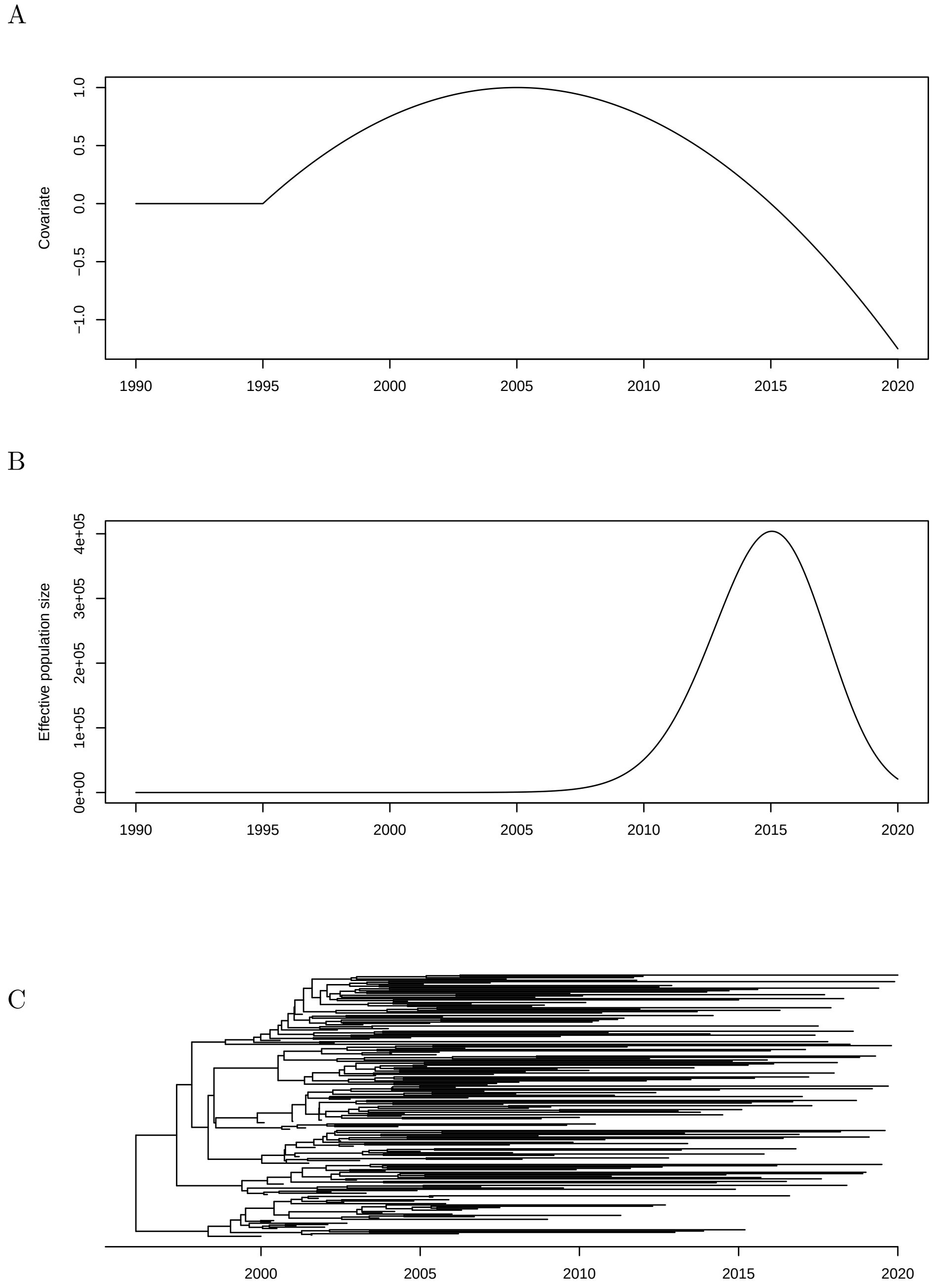

Figure S11: Example of simulation with covariate data driving the growth rate. (A) Covariate data. (B) Effective population size. (C) Dated phylogeny.

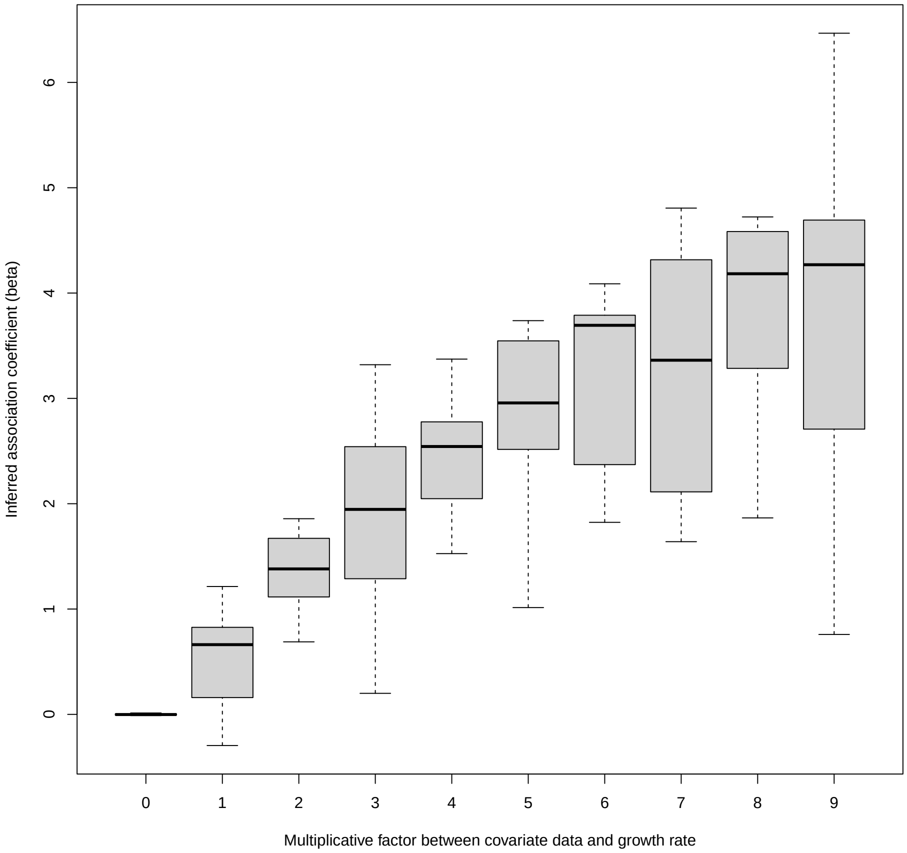

Figure S12: Results of the covariate analysis. For each value of the Gaussian noise (x-axis) ten simulations were performed and the inferred values of the association coefficient β are shown (y-axis) as boxplots.
